# Adaptation of Proteins to the Cold in Antarctic Fish: A Role for Methionine?

**DOI:** 10.1093/gbe/evy262

**Published:** 2018-11-29

**Authors:** Camille Berthelot, Jane Clarke, Thomas Desvignes, H William Detrich, Paul Flicek, Lloyd S Peck, Michael Peters, John H Postlethwait, Melody S Clark

**Affiliations:** 1 Laboratoire Dynamique et Organisation des Génomes (Dyogen), Institut de Biologie de l'Ecole Normale Supérieure – UMR 8197, INSERM U1024, Paris Cedex 05, France; 2 European Molecular Biology Laboratory, European Bioinformatics Institute, Cambridge, United Kingdom; 3 Department of Chemistry, University of Cambridge, United Kingdom; 4 Institute of Neuroscience, University of Oregon; 5 Department of Marine and Environmental Sciences, Marine Science Center, Northeastern University; 6 British Antarctic Survey, Natural Environment Research Council, Cambridge, United Kingdom

**Keywords:** protein folding, gene duplication, positive selection, map kinases, environmental stress response, reactive oxygen species

## Abstract

The evolution of antifreeze glycoproteins has enabled notothenioid fish to flourish in the freezing waters of the Southern Ocean. Whereas successful at the biodiversity level to life in the cold, paradoxically at the cellular level these stenothermal animals have problems producing, folding, and degrading proteins at their ambient temperatures of –1.86 °C. In this first multi-species transcriptome comparison of the amino acid composition of notothenioid proteins with temperate teleost proteins, we show that, unlike psychrophilic bacteria, Antarctic fish provide little evidence for the mass alteration of protein amino acid composition to enhance protein folding and reduce protein denaturation in the cold. The exception was the significant overrepresentation of positions where leucine in temperate fish proteins was replaced by methionine in the notothenioid orthologues. We hypothesize that these extra methionines have been preferentially assimilated into the genome to act as redox sensors in the highly oxygenated waters of the Southern Ocean. This redox hypothesis is supported by analyses of notothenioids showing enrichment of genes associated with responses to environmental stress, particularly reactive oxygen species. So overall, although notothenioid fish show cold-associated problems with protein homeostasis, they may have modified only a selected number of biochemical pathways to work efficiently below 0 °C. Even a slight warming of the Southern Ocean might disrupt the critical functions of this handful of key pathways with considerable impacts for the functioning of this ecosystem in the future.

## Introduction

One of the many consequences of our warming world will be the irretrievable loss of the coldest habitats. This change will have a significant impact on the endemic fauna, many of which have evolved novel adaptations to life in freezing conditions (Portner et al. 2007). Clearly, there is strong interest in learning how these cold-adapted species will respond in the coming years, in particular with regard to those species of economic importance such as the notothenioid fish in the Southern Ocean. We are constrained in our abilities to decipher responses to change, however, because we know relatively little about the molecular genetic mechanisms these species have evolved to thrive in freezing conditions and how these adaptations will impact their future responses and resilience in a changing world.

Rapid climate change is affecting oceans in both polar regions, more specifically the Arctic and along the Antarctic Peninsula (Abram et al. 2013; Nicolas and Bromwich 2014). Although both regions contain freezing seas, their faunas have different evolutionary histories based on geography (Hunt et al. 2016). The North Pole is in the open ocean and Arctic benthic fauna are largely a product of the Pleistocene glaciation (2.58 Myr) and with few endemic species (Dunton 1992). In comparison, the break-up of Gondwana resulted in the South Pole being in the middle of a continent. The opening of the Drake Passage (25–22 Myr) between South America and Antarctica, and the subsequent development of the Antarctic Circumpolar Current around the continent and the Antarctic Polar Front (Maldonado et al. 2014; Scher et al. 2015) effectively isolated the marine fauna around Antarctica. The Southern Ocean gradually cooled with the formation of sea ice 14–12 Myr (Shevenell et al. 2011). The resident fauna has, therefore, been subjected to long and intense selective pressure for survival in freezing waters, which is accompanied with high levels of oxygenation and therefore heightened exposure to reactive oxygen species (ROS) (Abele and Puntarulo 2004). In general, Antarctic species are characterized by long development and generation times, deferred maturity and extended life spans (Peck 2018); thus, a key factor to which they are not adapted is rapid environmental change.

These evolutionary pressures of isolation and cooling in the Southern Ocean led to local extinction of most fish fauna but a massive radiation of the notothenioid fish, which are classed as a rare example of a marine species flock (Lecointre et al. 2013). Of 129 catalogued notothenioid species, 101 are present only in the Southern Ocean (Eastman and Eakin 2000), and key members of this order are targets for commercial fisheries. Notothenioids have developed some distinct adaptations to life in the cold and the associated characteristic of highly oxygenated waters (Carpenter 1966; Peck 2018). These cellular adaptations include the possession of antifreeze glycoproteins (Devries 1971; Chen et al. 1997; Near et al. 2012), cold-stable yet dynamic cytoplasmic microtubules (Detrich et al. 1989; Himes and Detrich 1989; Billger et al. 1994; Detrich et al. 2000), and giant muscle fibers with reduced fiber numbers (Johnston et al. 2003). One family, the Channichthyidae (commonly known as the icefish), are highly derived. They lack hemoglobin and functional erythrocytes, and several icefish species are devoid of myoglobin in both cardiac and skeletal muscle (Sidell and O’Brien 2006).

Whereas protein denaturation at high temperatures has long been recognized, there is less appreciation that proteins denature in response to cold stress (Privalov 1990; Peck 2016). At the molecular level, a number of studies have identified amino acid changes that increase the flexibility of some Antarctic proteins, allowing them to work more efficiently around 0 °C. Although the number of amino acid substitutions in each of the Antarctic fish proteins was small when compared with their temperate orthologues, they generally involved the replacement of charged or large hydrophobic amino acids with smaller nonpolar residues in regions of the protein that increased molecule flexibility and catalytic efficiency. Examples of these include lactate dehydrogenase (A_4_-LDH), chaperonin containing TCP-1 (CCT), α- and β-tubulins, l-glutamate dehydrogenase, and pepsin A2, etc. (Fields and Somero 1998; Detrich et al. 2000; Ciardiello et al. 2000; Carginale et al. 2004; Pucciarelli et al. 2006). Other results have shown that retention of gene duplicates by cold-living species helps to ensure that protein production levels are maintained at levels comparable to temperate species, albeit using twice the number of genes (Carginale et al. 2004; de Luca et al. 2009). Further reports indicate that protein production, unfolding, and accumulation of ubiquitinated proteins may be significant problems in Antarctic species (Peck 2016, 2018). Antarctic fish contain higher levels of ubiquitin-tagged proteins than closely related temperate species and constitutively express the “inducible” form of the 70-kDa heat shock protein (HSP70), a molecular chaperone that helps to rescue denatured proteins (Hofmann et al. 2000; Place et al. 2004; Place and Hofmann 2005; Clark et al. 2007; Todgham et al. 2007, 2017). Thus, steady-state protein production and functioning is likely to be much less efficient in Antarctic species compared with temperate relatives, despite the protein adaptations detailed above. Identification of the extent to which the proteome of Antarctic notothenioid fish is cold-adapted should provide critical information for predicting how these species will respond to a changing world. Fortunately, our ability to tackle these questions by interrogating protein amino acid sequences for cold-adapted substitutions is improving rapidly.

Since the original Antarctic toothfish *Dissostichus mawsoni* study on Expressed Sequence Tags (ESTs) (Chen et al. 2008) and the emergence of Next Generation Sequencing, molecular data for notothenioids has gradually increased. These data have largely been obtained using Roche 454 with published studies often restricted to a single species (Windisch et al. 2012; Shin et al. 2012; Coppe et al. 2013; Papetti et al. 2015). To date, only head kidney tissue has been subjected to short read Illumina sequencing (Gerdol et al. 2015), with a mixed approach used to generate the first draft genome of the bullhead notothen *Notothenia coriiceps* (Shin et al. 2014). Three studies have developed preliminary transcriptomes with the aim of identifying notothenioid responses to warming (Huth and Place 2013; Bilyk and Cheng 2013, 2014), but given the piecemeal information available with regard to tissues, treatments and different sequencing platforms, it is difficult to directly compare results across studies to generate an unbiased global overview of notothenioid gene evolution to the cold at the amino acid level.

**Fig. 1.**
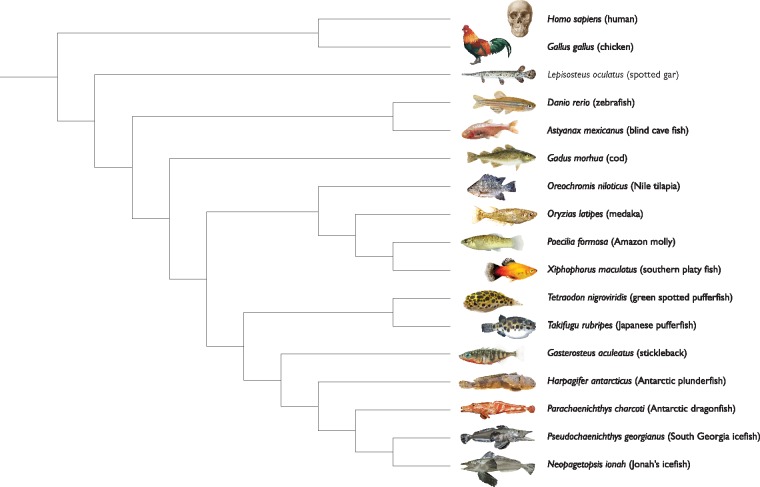
—Phylogenetic placement of notothenioid fish to demonstrate relatedness to other species used in these analyses and for the positive selection scans. On the basis of Near et al. (2012) and Betancur et al. (2013).

In this study, we sequenced the transcriptomes of four Antarctic notothenioids using high-throughput sequencing. These species included two icefish (*Neopagetopsis ionah* [Jonah’s icefish] and *Pseudochaenichtys georgianus* [South Georgia icefish]) and two red-blooded species (*Harpagifer antarcticus* [Antarctic spiny plunderfish] and *Parachaenichthys charcoti* [Charcot’s dragonfish]) (fig. 1). These species are phylogenetically distinct within the notothenioids, being taxonomically assigned to three families (Channichthyidae, Harpagiferidae, and Bathydraconidae) and four genera (Near et al. 2012). These species choices enabled us to differentiate between generalized notothenioid-specific changes in amino acid composition and those that are species-specific in comparison to orthologous sequences from temperate teleost relatives. These global analyses are described and discussed in the context of cold-adaptation and the hypothesis that Antarctic fish proteomes are incompletely adapted to function efficiently in freezing oceans.

## Materials and Methods

**Fig. 2.**
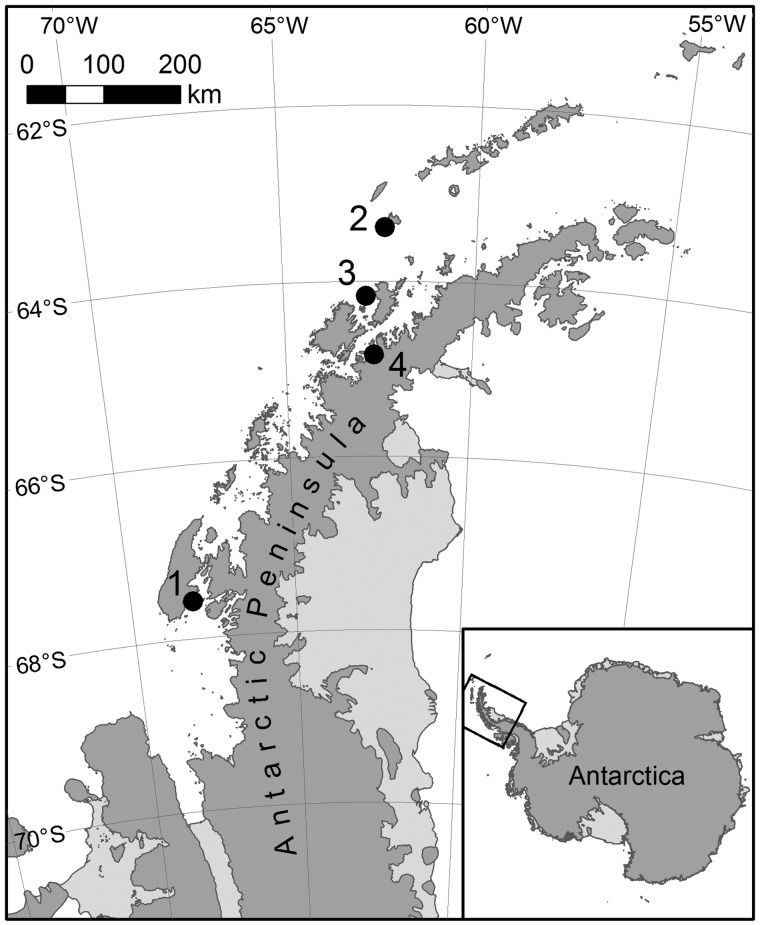
—Wild capture locations for the notothenioid fish samples used in this study. Numbers correspond to the following locations: 1: Ryder Bay, 2: south of Low Island (Antarctic Specially Protected Area No. 152, Western Bransfield Strait), 3: west of Brabant Island (Antarctic Specially Protected Area 153, Eastern Dallmann Bay) in the Palmer Archipelago, 4: Andvord Bay near Palmer Station.

*Material:* *Harpagifer* *antarcticus* (Nybelin, 1947) were collected by divers from a depth of 12 m from Ryder Bay (67°34′07″S, 68°07′30″W), close to the Rothera research station run by the British Antarctic Survey on the Antarctic Peninsula. Seven tissues were dissected and flash frozen in liquid nitrogen for subsequent extraction of RNA. Specimens of the dragonfish *P.charcoti* (Vaillant, 1906) and of the South Georgia icefish *P.georgianus* (Norman, 1937) were collected by bottom trawls deployed from the *ARSV Laurence M. Gould* south of Low Island (Antarctic Specially Protected Area No. 152, Western Bransfield Strait) or west of Brabant Island (Antarctic Specially Protected Area 153, Eastern Dallmann Bay) in the Palmer Archipelago (March–June, 2012 and 2013). Fourteen and ten tissues were dissected, respectively from each species and flash frozen in liquid nitrogen for subsequent extraction of RNA. A single specimen of the icefish *N.ionah* (Nybelin, 1947) was captured by bottom trawling in Andvord Bay (May, 2012) near Palmer Station (fig. 2). Only spleen tissue was available from this species (supplementary table S1, Supplementary Material online). The latter was included to ensure representation of a second ice fish species in the transcriptome data set.

### Transcriptome Sequencing and Assembly

Total RNA was extracted from 58 samples across the four species using TRIsure (Bioline) according to manufacturer’s instructions and purified on RNeasy columns (Qiagen). Quantity and quality were checked on an Agilent TapeStation 2200. The concentrated RNA samples were submitted to the EMBL GeneCore facility (Heidelberg, Germany) and used to generate barcoded normalized cDNA libraries with an average fragment length of ∼130 bp. The libraries were multiplexed and sequenced over four lanes on a HiSeq2000 platform (Illumina, San Diego, USA) using 100 bp paired-end reads. Read quality was assessed using FastQC (http://www.bioinformatics.babraham.ac.uk/projects/fastqc/; Last accessed September 2015). For each of the four species, raw sequencing reads from the different tissues (supplementary table S1, Supplementary Material online) were pooled for full transcriptome assembly performed with Trinity (Grabherr et al. 2011), using Trimmomatic (Bolger et al. 2014) to preprocess and clip low-quality reads and remaining adapter sequences, and default parameters otherwise.

### Transcriptome Quality Control

Reads used for the de novo assemblies were remapped to the assembled transcripts using Bowtie (Langmead et al. 2009) with the parameters implemented in the RSEM pipeline (Li and Dewey 2011). Transcriptome completeness was estimated with BUSCO using the *Eukaryota*, *Metazoa*, *Vertebrata*, and *Actinopterygii* reference gene sets (Simão et al. 2015).

### Orthology Assignments

For sequence evolution analyses, transcripts were additionally assigned to orthologous gene families based on three-way orthologous relationships with stickleback (*Gasterosteusaculeatus*) and zebrafish (*Daniorerio*). Curated, nonredundant cDNA sets for stickleback and zebrafish were downloaded from Ensembl v81 (Cunningham et al. 2015) and compared with the four notothenioid transcriptome assemblies independently using BlastX to identify putative orthologs (–max_target_seqs 1, –evalue 1e–40) (Altschul et al. 1990). Family assignation was considered robust when the stickleback and zebrafish transcripts separately identified by the BlastX analysis of the four notothenioid transcriptome assemblies themselves corresponded to a pair of orthologous genes according to the Ensembl Compara gene trees (Vilella et al. 2008). Transcripts that could not be assigned an orthologue in either of the two species, or resulted in a discrepancy between stickleback and zebrafish (i.e., the best BLAST hits were identified as paralogous in the Ensembl Compara gene trees, or otherwise nonorthologous genes) were not used for sequence evolution analyses. The OMA pipeline was also run with default parameters on the four notothenioid transcriptomes to independently identify orthologues across notothenioids (Altenhoff et al. 2015). Orthology groups were considered as consistent when a group of orthologous notothenioid transcripts identified by one method fully overlapped the group identified by the other and were kept for analyses.

### Sequence Alignments

Once transcripts were assigned to an orthologous gene family, the Ensembl API was used to retrieve the orthologous cDNA sequences in all 11 fish available in the database (*D.rerio* [Zebrafish], *G.aculeatus* [Stickleback], *Astyanax mexicanus* [Cavefish], *Gadus morhua* [Cod], *Oreochromis niloticus* [Tilapia], *Oryzias latipes* [Medaka], *Xiphophorus maculatus* [Platyfish], *Poecilia formosa* [Amazon molly], *Takifugu rubripes* [Fugu or the Japanese pufferfish], *Tetraodon nigroviridis* [Tetraodon or the green spotted pufferfish], *Leipisteus oculatus* [Spotted gar]), as well as human (*Homo sapiens*) and chicken (*Gallus gallus*) as outgroups. The orthologous transcripts were translated to protein sequences and the protein sequences aligned using T-Coffee with default parameters (Notredame et al. 2000). The alignments were then back-translated to the transcript sequences using the “backtrans” utility from TreeBeST (Vilella et al. 2008). Low-quality blocks in the sequence alignments were removed using GBlocks (–t = c, –b5 = h) (Castresana 2000). Gene trees reconciled with the species phylogeny were built using TreeBeST v.1.9.2 with default parameters.

### Analyses of Amino Acid Composition and Substitutions

Global amino-acid composition was computed across species on translated orthologous transcripts to ensure that missing or redundant transcripts in the notothenioid transcriptomes would not skew the comparisons. High-quality blocks were extracted from the protein alignments using the “seq_reformat” utility in T-Coffee (minimum column score of 8). The filtered alignments were then parsed using a custom Python script to identify sites where notothenioids exhibit the same amino-acid substitution compared with reference temperate fish (stickleback and zebrafish), that is, a presumably fixed substitution that occurred in the ancestral notothenioid branch.

### Positive Selection Scans

Codeml (Yang 2007) was run on the sequence alignments and reconciled gene trees (described above) using the *ETE3* Python package (Huerta-Cepas et al. 2016) to detect proteins with signs of either positive selection or constraint relaxation specifically in Notothenioids. Synonymous and nonsynonymous substitution rates were estimated using branch models with the ancestral Notothenioidei branch as the foreground and other branches in the tree as the background (null model “M0,” free evolution of the foreground “b_free,” and neutral evolution of the foreground “b_neut”). The two nonnull models were compared with the null model for each tree using a likelihood ratio test.

## Results

### Transcriptome Assemblies

To document the protein-coding gene content of notothenioid genomes, we performed mRNA-seq on one to four individuals for each of the four species included in the study. Samples were collected from animals captured in Ryder Bay, south of Low Island, west of Brabant Island and in Andvord Bay during field trips in Antarctica in 2013 and 2014 (fig. 2). Tissue collection was driven by availability and included one to thirteen tissues per individual (supplementary table S1, Supplementary Material online). As a result, the obtained transcriptomes varied in quality and completeness across species: *P. georgianus* and *P. charcoti* were the most exhaustively sampled, whereas *N. ionah* was only represented by one tissue (spleen) from one individual. The latter was included to ensure representation of a second ice fish species in the transcriptome data set. Sequencing yielded from 8 to 39 million paired-end reads per library, which constituted a total of 14–503 million read pairs per species.

**Fig. 3.**
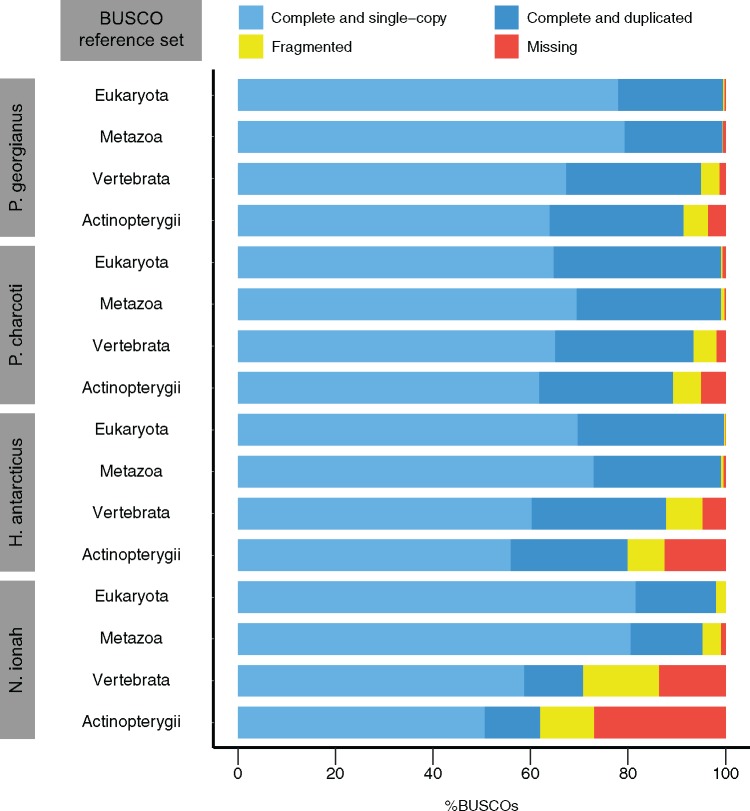
—Completeness assessment of the four de novo notothenioid transcriptome assemblies using the BUSCO (Simão et al. 2015) reference gene sets. The reference sets include 303, 978, 2,586, and 4,584 genes for *Eukaryota*, *Metazoa*, *Vertebrata*, and *Actinopterygii*, respectively.

Transcriptomes were assembled de novo using Trinity (Grabherr et al. 2011) on quality-filtered pooled reads from all individuals and tissues for each notothenioid species (Materials and Methods). The assemblies contained 72,573–251,528 transcripts across species, grouped into 61,696–212,979 “gene” units by Trinity (table 1). Median contig length was ∼400 bp and was consistent across species. Over 80% of initial reads could be realigned to the assembled transcriptomes, which is consistent with high assembly quality (Haas et al. 2013). We then assessed transcriptome completeness using the Benchmarking Universal Single-Copy Orthologs (BUSCO) method (Simão et al. 2015). This analysis confirmed that the great majority of single-copy genes found across all vertebrate and Actinopterygian species were present and complete in our assemblies (fig. 3). As expected, the *N. ionah* transcriptome from a single tissue was less exhaustive than for the other three species; nonetheless, 62% of reference Actinopterygian genes were successfully identified as full-length transcripts in this species.

**Table 1:** De Novo Assembly and Annotation Statistics for the Four Notothenioid Transcriptomes Included in This Study

	*P. georgianus*	*P. charcoti*	*H. antarcticus*	*N. ionah*
Number of sampled individuals	4	2	1	1
Number of sampled tissues	10	13	6	1
Total number of mRNA-seq libraries	28	23	6	1
Total pairs of paired-end reads	503,200,884	368,989,424	89,734,790	14,297,063
Number of transcripts in de novo assembly	251,528	173,461	145,576	72,573
Number of “gene” units	212,979	144,437	118,095	61,696
Median transcript length (bp)	374	390	408	442
Mean transcript length (bp)	822	914	890	874
N50 (bp)	1,725	2,032	1,827	1,624
Total assembled bases	206,860,807	158,515,494	129,565,375	63,417,105
Fraction of reads mapping to transcripts	83.8%	80.0%	80.7%	81.7%
Number of transcripts with temperate orthologs	26,072	26,974	24,963	16,941
Number of genes in orthology groups	10,728	10,728	8,611	7,093

Note.—“Gene” units correspond to Trinity's definition of a gene, that is, a group of transcripts that likely are isoforms from the same gene.

### Identification of Orthologous Genes

We used stickleback (*G.aculeatus*) and zebrafish (*D.rerio*) as high-quality temperate teleost reference genomes to identify and annotate orthologous sequences across notothenioid transcriptomes. Stickleback is the species with a sequenced genome that is both most closely related to notothenioids (fig. 1) and for which there is good annotation and functional validation of gene roles. Because our proposed analyses relied on accurate rather than exhaustive orthology identification, we used a fairly conservative approach. Briefly, notothenioid transcripts were compared with stickleback and zebrafish transcriptomes using BLAST (Altschul et al. 1990) with stringent e-value thresholds to identify the top match as a putative orthologue in each reference species (Materials and Methods). We considered the orthology relationship as high-confidence when the putative orthologues in stickleback and zebrafish correspond to an orthologous gene pair in the Ensembl database (Vilella et al. 2008; Herrero et al. 2016). We successfully identified 16,941–26,974 transcripts with high-confidence orthologues in temperate fish across notothenioid transcriptomes (table 1). This process resulted in the identification of 10,728 fully consistent orthology groups present in stickleback, zebrafish and at least two of the notothenioid fish (*P. georgianus* and *P. charcoti*). As a control, we also annotated orthology groups across notothenioid transcriptomes using the OMA pipeline (Altenhoff et al. 2015): 8,697 orthology groups (81%) were consistent with the conservative groups obtained when anchoring the analysis on the stickleback and zebrafish gene sets.

### Amino Acid Usage in Notothenioid Proteins

**Fig. 4.**
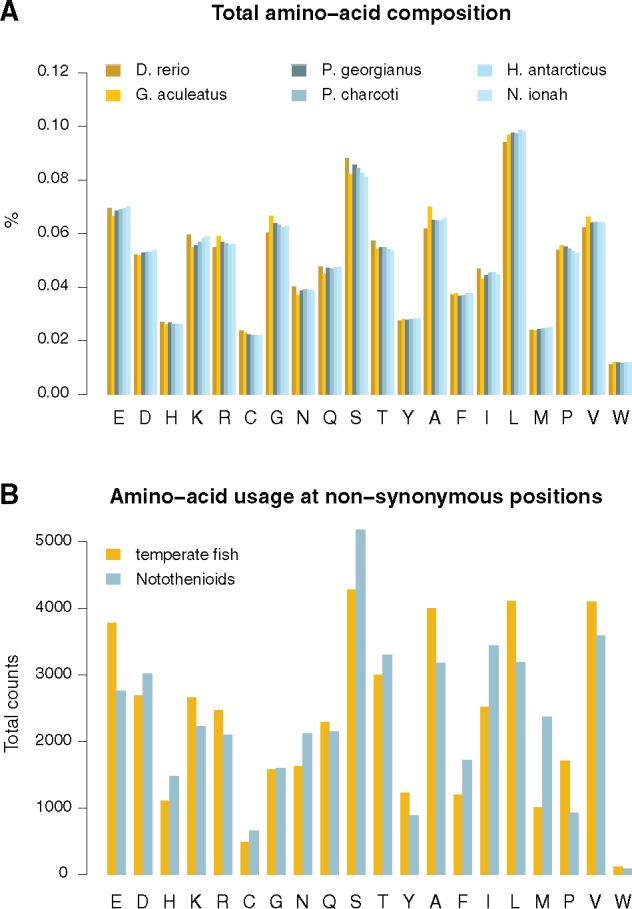
—Amino-acid usage in temperate and notothenioid fish proteins. (*A*) Total amino-acid composition deduced from the translated sequence of 10,728 orthologous transcripts across species. (*B*) Amino-acid usage at 45,994 reliable nonsynonymous positions between temperate and notothenioid fish proteins.

**Fig. 5.**
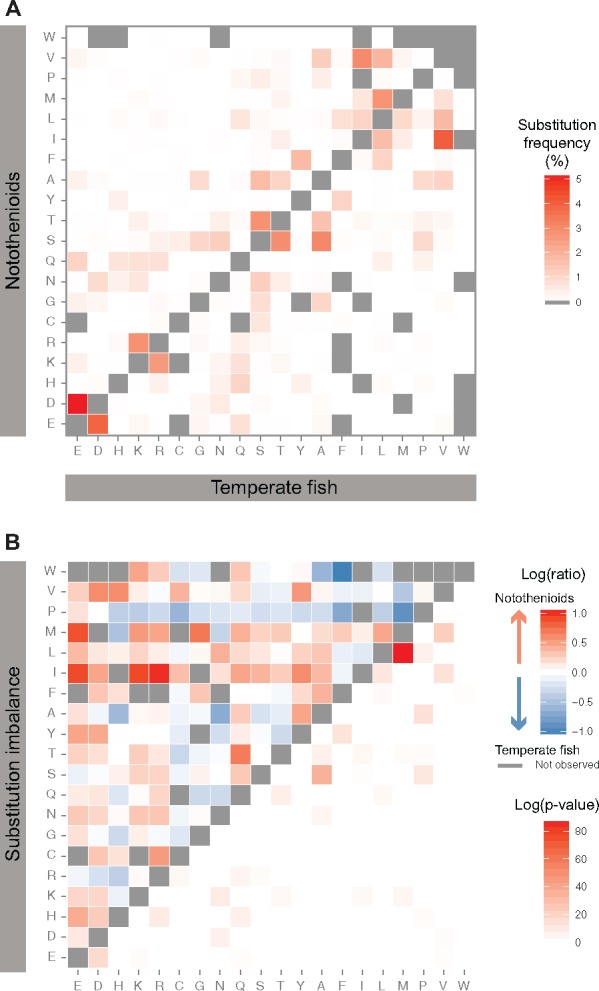
—Amino-acid usage imbalance at nonsynonymous positions between temperate and notothenioid fish proteins. (*A*) Substitution frequencies at 45,994 reliable nonsynonymous positions between temperate and notothenioid fish. Percentages are expressed as the fraction across all observed substitutions. Substitutions that were never observed in the curated data sets are shown in grey. (*B*) Top left part of the heat map represents the imbalance in the substitution frequencies for any pair of amino-acids between temperate and notothenioid fish. Substitutions favored in notothenioids are shown in red, whereas substitutions depleted in notothenioids are shown in blue. Substitutions that are never observed in at least one direction are shown in grey. Bottom right part of the heat map represents the *P*-value of the proportion test (FDR-corrected, Benjamini–Hochberg adjustment).

We sought to examine whether notothenioid proteins exhibited preferential amino-acid usage compared with temperate fish and whether usage differences aligned to the classically accepted psychrophilic modifications in bacteria, such as a decrease in proline, arginine and aromatic residues (Struvay and Feller 2012; Yang et al. 2015). Overall, amino acid compositions inferred from the whole sequence across the 10,728 orthology groups showed no differences between the four notothenioids and the two temperate control fish (stickleback and zebrafish; fig. 4*A*). We then focused on 45,994 amino acid residue positions where all four notothenioids showed a concordant substitution compared with the amino acid present in temperate fish. These positions were of interest because they likely represented substitutions that occurred during the evolution of notothenioids and were highly unlikely to have resulted from errors in the transcriptome assemblies. At these positions, the Antarctic fish tended to favor methionine (M), isoleucine (I), phenylalanine (F) and serine (S), and disfavor proline (P), glutamic acid (E), leucine (L), and alanine (A) (fig. 4*B*; Chi^2^ test, Bonferroni-correct *P*-value cutoff < 10^−15^). The biased usage of serine and methionine seems to be a derived characteristic of notothenioids, because a comparison to more distant human and chicken orthologues yielded similar observations (supplementary fig. S1, Supplementary Material online). A series of biased amino acid substitutions have taken place in the notothenioids, as shown by the red boxes in figure 5*A*. When these substitutions were tested for enrichment, ratio tests showed an imbalance in methionine, isoleucine, lysine (K), and arginine (R) (red boxes, upper most section of fig. 5*B*). While some ratios were skewed due to low numbers, the most significant bias was the overrepresentation of positions where leucine in the temperate fish was replaced by a methionine in the notothenioids (lower section of fig. 5*B*). However, we could not find evidence that these proteins were evolving under positive selection based on comparisons of synonymous versus nonsynonymous substitution rates so the evolutionary impact of those amino-acid substitutions remains unclear.

## Discussion

The work presented here provides the first comprehensive comparison of the transcriptomes of multiple Antarctic notothenioids from different taxa with respect to temperate species, thereby negating false positives due to species-specific evolution. The most notable result was the significant overrepresentation of positions where leucine in the other fish had been replaced by methionine (M, codon AUG) in the notothenioids (fig. 5, bottom heat map). Detailed analyses at concordant, nonsynonymous positions between notothenioids and temperate fish showed that Antarctic fish in addition to the significant bias in methionine also favored serine (S, codons UCN and AGU/C) and isoleucine (I, codons AUU/C/A) over leucine (L, codons CUN), glutamic acid (E, codons GAA/G), lysine (K, codons AAA/G), and arginine (R, codons CGN and AGA/G) (figs. 4 and 5). Similar results for serine and glutamic acid have been reported previously in the Antarctic zoarcid *Pachycara brachycephalum* (Windisch et al. 2012), but the isoleucine and methionine results are novel to this study. The reason behind this significant bias towards methionine remains unclear, especially because both leucine and methionine are nonpolar, hydrophobic amino acids and this is not a substitution identified in previous cold adaptation work in bacteria and Archaea (cf., Yang et al. 2015). For such a change to occur, in most instances two nucleotide mutations would be required in the triplet code to convert leucine (CUU, CUC, CUA, CUG, UUA, or UUG) into methionine (AUG), which would be a rare event. Methionine residues are rare in vertebrate proteins (<2%) and are normally quite conserved.

Therefore, we hypothesize that these “extra” methionines are involved in redox regulation. The freezing, oxygen-rich waters of the Southern Ocean promote the formation of ROS and would be expected to lead to enhanced ROS damage of DNA and membrane lipid peroxidation in polar species (Abele and Puntarulo 2004). This may be particularly critical in the notothenioids, which have increased mitochondrial densities, have more polyunsaturated lipids and use lipids as a primary energy source (Todgham et al. 2017). To promote the protection of the genome, particularly in long-lived species like notothenioid fish (Buttemer et al. 2010), the expectation would be the replacement of methionines with leucines, or similar amino acids because the sulfur-containing amino acids (methionine and cysteine) are targets for ROS. Indeed a reduction in methionines has been identified in psychrophilic prokaryotes when compared with thermophilic species (Yang et al. 2015). In mammals, however, these sulfur-containing amino acids have been shown to act as critical antioxidant regulators (Stadtman et al. 2002). Both methionine and cysteine can undergo reversible modifications to modulate physiological protein functions and acting as molecular redox switches (Pajares et al. 2015). As supporting evidence for this hypothesis, studies on two Antarctic bivalve molluscs (*Aequiyoldia eightsi*, previously *Yoldia eightsi*) and (*Adamussium colbecki*) have shown trade-offs between the thermal tolerance and antioxidation potential of the animal (Regoli et al. 1997; Abele et al. 2001). In both instances, the Antarctic antioxidant enzymes catalase and superoxide dismutase (SOD) are highly efficient at 0 °C, more so than the orthologous enzymes in closely related temperate relatives, but these Antarctic enzymes lose their activity with even small increases in temperature (Regoli et al. 1997; Abele et al. 2001). The resulting hypothesis was that at least some of the enzymes involved in the Antarctic bivalve antioxidant system have been fine-tuned to work at freezing temperatures. Recent work in Antarctic fish examined antioxidant activity in three species related to warming (Enzor and Place 2014). At the end of the two month experiment levels of cellular damage, as measured by protein carbonyls, were still slightly above those of control animals, indicating incomplete acclimation to 4 °C after 56 days. This may indicate potential trade-offs occurring at the cellular level and incomplete compensation to chronic oxidative damage or damage from other sources (Enzor and Place 2014). Combatting ROS in these fish species is clearly important as other transcriptome analyses have shown enrichment for genes involved in anti-oxidation (Chen et al. 2008; Shin et al. 2012; Bilyk and Cheng 2013).

The results described here for notothenioid fish differ from other studies on psychrophiles. In prokaryotic studies specific amino acid substitutions have been associated with cold adaptation, including a reduced use of proline (P), arginine and acidic amino acids in bacteria, a lower content of hydrophobic amino acids (leucine in particular) in the Archaea and a higher content of noncharged polar amino acids, especially glycine (G) and threonine (T) in bacteria (Saunders et al. 2003; Ayala-del-Rio et al. 2010; Zhao et al. 2010; Yang et al. 2015). These changes result in increased flexibility when compared with mesophilic proteins, especially of the active site, which is deemed essential for efficient enzyme functioning in the cold (Struvay and Feller 2012). Although similar types of changes have been identified in individual Antarctic fish proteins (Fields and Somero 1998; Detrich et al. 2000; Ciardiello et al. 2000; Carginale et al. 2004; Pucciarelli et al. 2006), these are not evident, in terms of biased amino acid substitutions, from the global analyses presented here. However, it is still not possible to predict a priori with any great accuracy how particular amino acid substitutions will affect protein activity at different temperatures (Fields et al. 2015). Although increasingly sophisticated algorithms are being developed to predict protein activity in response to temperature changes and protein stability following mutations, accurate prediction of reaction rates of enzymes across a temperature gradient remains difficult, not least because all interactions are context-dependent (Fields et al. 2015; Pucci and Rooman 2017). In many cases, alteration in enzyme performance can result from a single amino acid substitution which can be demonstrated only by functional analyses (Fields et al. 2015). The situation is further complicated by the fact that different proteomic amino acid compositional motifs have been demonstrated between archaea, eubacteria and eukaryotes (Pe’er et al. 2004), which may partly explain why Antarctic fish display different amino acid substitutions than those identified in bacteria and Archaea.

It is now clear from prokaryotic analyses that, in terms of amino acid composition, low temperature adaptation is not necessarily the opposite of high temperature adaptation with kinetic stabilization against cold denaturation suggested as a cold adaptation mechanism (Romero-Romero et al. 2011; Yang et al. 2015). However, with the vast majority of data underpinning these observations originating from single celled organisms (especially bacteria) (cf., Methe et al. 2005; Struvay and Feller 2012; Yang et al. 2015; Pucci and Rooman 2017) it may well be that the situation is more complex in psychrophilic multicellular organisms, in which there is a relative paucity of both sequence data and functional analyses.

Only a handful of protein adaptations have been functionally described in Metazoa living at subzero temperatures. Published studies of some Metazoa do, however, include some of the adaptations detailed above leading to the evolution of proteins with higher maximum activity and lower temperature of maximum stability, for example, lactate dehydrogenase, tubulins, l-glutamate dehydrogenase, the transmembrane protein *Sec61*, and the TCP chaperone complex in Antarctic fish (Fields and Somero 1998; Detrich et al. 2000; Ciardiello et al. 2000; Romisch et al. 2003; Pucciarelli et al. 2006; Cuellar et al. 2014) as well as the evolution of novel variants such as nothepsins in the aspartate protease family (De Luca et al. 2009). For some other genes (e.g., tubulins and pepsins) relative protein production levels in notothenioids are similar to those in temperate species due to gene duplication events in Antarctic species, where two genes produce similar amounts of protein as one gene in temperate relatives (Parker and Detrich 1998; Carginale et al. 2004). Transcriptomic studies in notothenioids have also detailed additional gene duplication events, particularly for transcripts involved in response to oxidative stress, but these lack characterization at the protein level and evaluation of catalytic functions (cf., Chen et al. 2008; Windisch et al. 2012; Coppe et al. 2013).

To date, the vast majority of analyses of Antarctic fish genes, including the one described here, is largely constrained by the fact that the interrogated data sets are all transcriptomes. Many transcriptome studies focus on either single species, or a limited number of tissues. Additionally, many of these studies compare a single species of Antarctic fish with a single temperate relative and therefore cannot avoid any potential lineage-specific, species-specific or tissue-specific biases. Analysis of the amino acid substitutions in several candidate proteins emphasized the need to study such changes across multiple species. Three selected proteins studied here (calmodulin, neuroglobin and SOD1, supplementary material S1, Supplementary Material online), showed more species-specific amino acid substitutions within notothenioids than between notothenioids and other fish species. In addition, the positions and types of proposed “notothenioid-specific” changes were invariably in the same coding sites that showed high variability among other fish species, with substitutions at the same position of up to six different amino acid residues with many different properties (supplementary material S1, Supplementary Material online). The question of how extensive cold-specific gene duplication events are in the notothenioids can only be answered with whole genome sequencing of multiple species. In addition, the extent to which these species use the different strategies of either amino acid substitutions to increase protein flexibility and/or gene duplication to increase protein quantity in the cold is unknown. The identification of such changes in proteins selected so far for functional studies may be serendipitous (e.g., most tubulins are notoriously cold-sensitive) and not follow the general rule.

The global analyses performed here provided little evidence for mass alteration of amino acid composition of Antarctic fish proteins (with the exception of methionines) that might have been predicted to enhance protein folding in the cold and reduce cold denaturation of protein, yet these fish are stenothermal (Somero and Devries 1967; Bilyk and Devries 2011). There is evidence that protein unfolding and accumulation of ubiquitinated proteins may be a significant problem in Antarctic species (Peck 2016, 2018). Analyses have shown that Antarctic fish contain higher levels of ubiquitin-tagged proteins than closely related temperate species (Place and Hofmann 2005; Todgham et al. 2007, 2017). In addition, Antarctic fish permanently express the inducible form of the HSP70, a molecular chaperone that targets denatured and unfolded proteins (Hofmann et al. 2000; Place et al. 2004; Clark et al. 2007). However, recent evidence suggests that the activity of the proteasome apparatus may be temperature-compensated in at least some tissues of notothenioid species (measured in *Trematomus bernacchii* and *Pagothenia borchgrevinki*) (Todgham et al. 2017). Gene expression analyses of the Ub-proteosome pathways suggests that this may be due to higher concentrations of proteasomes in the cell, although the catalytic efficiency of the 20S proteasome has yet to be evaluated (Todgham et al. 2017). Clearly this compensation is not sufficient to avoid the accumulation of unfolded proteins and the mechanism leading to this accumulation remains unknown. Thus, at the protein amino acid sequence level, these fish appear to be poorly adapted to the cold. This finding may explain the high levels of ubiquitination found in these species, but would not explain the stenothermality of these fish (Somero and Devries 1967; Bilyk and Devries 2011). These cellular results contrast with the ecological situation, whereby the notothenioids are highly successful. Future vulnerabilities will almost certainly be the result of complex interactions including the cellular level constraints detailed above and physiological and ecological characteristics such as very low metabolic rates, low energy lifestyles and limited food supply (Peck 2016, 2018).

## Summary

Even in bacteria, it is difficult using in silico approaches to define changes that enable a protein to function efficiently in the cold. It is also suggested that adaptation to the psychrophilic lifestyle is more the result of a suite of synergistic rather than unique changes (Methe et al. 2005). To date, bioinformatic approaches still have limited success in predicting function from sequence, hence there is a need to move towards high through-put testing of psychrophilic metazoan proteins to progress our understanding beyond that of a few isolated proteins and to develop an overview of genome-wide cold adaptations. The new era of long read sequencing will facilitate the generation of reference-quality genomes, and the notothenioids are currently one of the specialist groups targeted by the Sanger Institute as part of their contribution to the international Vertebrate Genome Project. These genomes will provide an invaluable resource for functional studies, which are clearly essential for understanding the subtle amino acid substitutions identified in this study, particularly the role arising from the significant increase in methionines. Given evidence accumulated to date, it is entirely possible that the Antarctic marine fauna are “clinging on” to life in their environment with only a selected number of biochemical pathways modified to work efficiently below 0 °C (Clark et al. 2017). In a warming world, it may be this relatively small number of cold-adapted proteins that are responsible for the vulnerability of these stenothermal species with considerable impacts for ecosystem functioning in the future.

## Supplementary Material

Supplementary data are available at *Genome Biology and Evolution* online.

## Supplementary Material

Supplementary Data
