# Supplementary material for: Adaptation of Proteins to the Cold in Antarctic Fish: A Role for Methionine?
Source: Genome Biol Evol. 2018 Nov 29;11(1):220–31. doi: 10.1093/gbe/evy262 (PMC6336007; doi:10.1093/gbe/evy262)
Supplement: Supplementary Data [file evy262_supp.pdf]

Supplementary Data

Adaptation of proteins to the cold in Antarctic fish: A role for Methionine?

Camille Berthelot[1,2], Jane Clarke[3], Thomas Desvignes[4], H. William Detrich, III[5], Paul Flicek[2], Lloyd S. Peck[6], Michael Peters[5], John H. Postlethwait[4], Melody S. Clark[6*]

[1]Laboratoire Dynamique et Organisation des Génomes (Dyogen), Institut de Biologie de l'Ecole Normale Supérieure - UMR 8197, INSERM U1024, 46 rue d'Ulm, 75230 Paris Cedex 05, France.
[2]European Molecular Biology Laboratory, European Bioinformatics Institute, Wellcome Genome Campus, Hinxton, Cambridge, CB10 1SD, UK.
[3]University of Cambridge, Department of Chemistry, Lensfield Rd, Cambridge CB2 1EW, UK.
[4]Institute of Neuroscience, University of Oregon, Eugene OR 97403, USA.
[5]Department of Marine and Environmental Sciences, Marine Science Center, Northeastern University, Nahant, MA 01908, USA.
[6]British Antarctic Survey, Natural Environment Research Council, High Cross, Madingley Road, Cambridge, CB3 0ET, UK.

***Corresponding Author:** Melody S. Clark, British Antarctic Survey, Natural Environment Research Council, High Cross, Madingley Road, Cambridge, CB3 0ET, UK. Email: mscl@bas.ac.uk

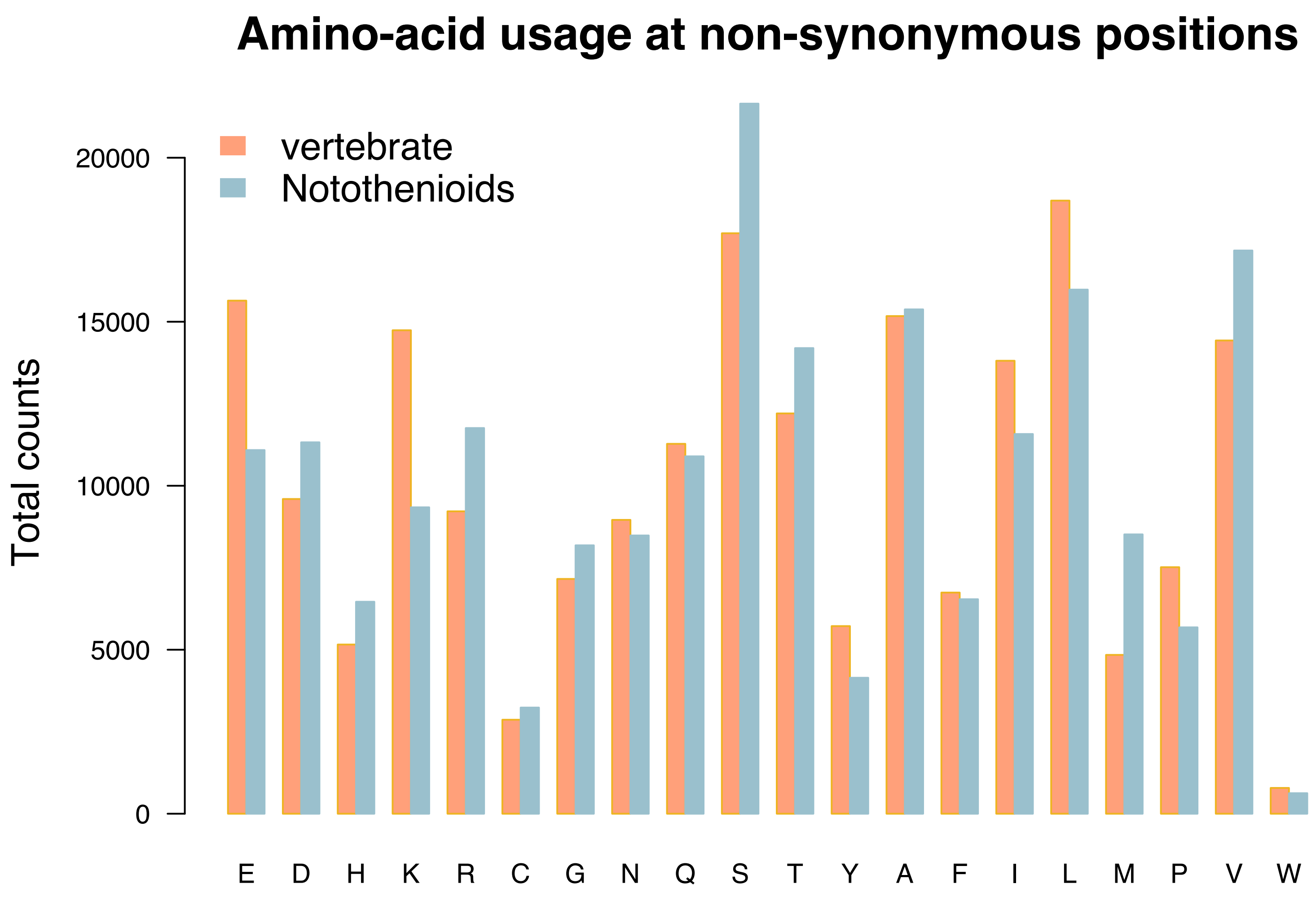

Amino-acid usage at non-synonymous positions
vertebrate
Notothenioids
Total counts
0
5000
10000
15000
20000
E
D
H
K
R
C
G
N
Q
S
T
Y
A
F
I
L
M
P
V
W

Supplementary Table S1: Number of raw reads produced for the different tissues from each of the four Notothenioids. In the cases where more than one animal was sampled, fish numbers 1-2 of *P. charcoti* correspond to the original fish IDs of Pcha2 and Pcha3, whilst fish numbers 1-4 of *P. georgianus* correspond to fish Ids Pgeo4, Pgeo7, Pgeo8 and Pgeo42013 respectively.

Species	Libraries	Sample number	Fish number			
			1	2	3	4
Neopagetopsis ionah	spleen	n=1	14297063			
Harpagifer antarcticus	Brain	n=1	12546421			
	White muscle	n=1	13377648			
	Liver	n=1	14256208			
	Kidney	n=1	17626116			
	Skin	n=1	11757830			
	heart	n=1	20170567			
Parachaenichtys charcoti	Brain	n=2	15799167	16587667		
	Ventricle	n=2	16583013	23591777		
	Pectoral muscle	n=2	14510454	16817765		
	White muscle	n=2	14104635	18283472		
	Liver	n=2	15994258	12042469		
	Spleen	n=2	19810431	15772144		
	Ovary	n=1	11340950			
	Testis	n=1		14027425		
	Head kidney	n=2	20526312	13890077		
	Trunk kidney	n=2	12090753	14346721		
	Whole blood	n=2	20190257	12797609		
	Intestine	n=1		18137550		
	Gill	n=1		14866598		
	Red blood cells	n=1		16877920		
Pseudochaenichtys georgianus	Brain	n=4	18331125	21409188	13818468	11886875
	Ventricle	n=3	39915788	11910723	18100509	
	Pectoral muscle	n=3	15339621	19639537	16514463	
	White muscle	n=3	16341220	18684208	8351606	
	Liver	n=3	32503701	21546986	31493263	
	Spleen	n=3	14895003	14613838	17384436	
	Ovary	n=2	15012348	15856491		
	Testis	n=1				11474215
	Head kidney	n=3	12843904	19601598	13214987	
	Trunk kidney	n=3	14928781	17621020	19966982	

Supplementary Information S1

Candidate genes: examples of amino acid changes in notothenioids

Methods

To evaluate notothenioid amino acid changes in a cross-section of genes, six candidates from a range of functional categories of interest were chosen for a more detailed examination. The selected genes comprised small, well-characterised genes present in multiple fish species (mainly teleosts, but in some cases these analyses were extended to the Actinopterygii when sequences from the spotted gar (*Lepisosteus oculatus*) were available) and available in at least two notothenioids. The amino acid composition of a series of six candidate genes were further analysed in depth at the amino acid sequence level. These genes were superoxide dismutase 1 (SOD1), neuroglobin, dihydrofolate reductase (both paralogues), p53 and calmodulin. Clustal X alignments were constructed from orthologues identified in the four Notothenioid transcriptomes along with other fish orthologues extracted from either SwissProt (Bateman et al., 2017) or Ensembl release 91 (Aken et al., 2017). Alignment data were visualised and annotated in BoxShade version 3.21 (https://embnet.vital-it.ch/software/BOX_form.html). Notothenioid-specific changes were noted and the type of substitution in terms of amino acid properties was noted.

Results

A range of amino acid changes were revealed, from virtually complete conservation between the notothenioids and other fish (calmodulin), to one amino acid substitution present in neuroglobin and SOD1, to more extensive changes in dihydrofolate reductase, dihydrofolate reductase-like and p53. In the case of the latter three genes, notothenioid-specific changes were at positions that were often highly variable in temperate fish species with up to six different amino acid substitutions and did not result in a definable pattern of directional substitution. In all cases, the notothenioid protein coding regions did not have consistent length differences compared to other fish.

Table showing amino acid substitutions in candidate proteins

Amino acid changes in Notothenioid candidate proteins compared with other fish. Colour code: blue: polar positive; red: polar negative; green: polar neutral; white: non-polar aliphatic; purple: non-polar aromatic; brown: small (proline and glycine); yellow: cysteine. Notes: 'variable' is recorded when substitutions in non-Notothenioid fish have 2-3 different amino acids with different properties in the same position; 'no significant change' is recorded where there is a change in amino acid in the notothenioids, which has the same physical properties as that of the non-notothenioid fish species.

Amino acid position	Notothenioid substitution	Substitutions in other fish		Notes
**Calmodulin**				
No Notothenioid-specific substitutions. 4 Notothenioid species-specific substitutions				
**Superoxide dismutase**				
6 Notothenioid species-specific substitutions				
198	Leu	Ile		No significant change
**Neuroglobin**				
3 Notothenioid species-specific substitutions. Last 13 aa at 3' end very different. 8 forms across 18 species.				
85	Pro	Thr		No significant change
**Dihydrofolate reductase**				

4 Notothenioid species-specific substitutions. Notothenioid 3’ end slightly longer by 2-6 aa								
4	Met	Val	Pro	Ile				No significant change
20	Asp	Asn	His	Lys				Negative to neutral/positive
22	Gln	Asn	Asp					No significant change
28	Val	Ile	Leu	Lys				No significant change
111	Thr	Ala	Val					Polar neutral to non-polar aliphatic
128	Gln	Glu	Gly					Variable
129	Phe	Ser	Arg	Ala	Gly			Variable
141	Met	Leu						No significant change
169	His	Gln	Glu	Leu	Val			Variable
**Dihydrofolate reductase-like**								
12 Notothenioid species-specific substitutions. First 10 aa highly variant 9 different forms in 11 species. Last 8 aa at 3’ end highly variant 9 forms in 11 species.								
28	Glu	Asp						No significant change
30	Gly	Gln	Arg	Thr	Ile			Variable
48	Thr	Arg	Gly	Ala	Ser			Variable
55	Phe	Met	Val	Lys				Non-polar to polar
78	Thr	Ser	Cys	Val	Ile			Variable
85	Leu	Lys	Arg	Thr				Non-polar aliphatic to polar
88	Asp	Glu	Phe	Ser	Thr	Ile	Gly	Variable
90	Pro	Val	Ala					No significant change
99	Ser	Glu	Gln	Gly	Lys	His		Variable
109	Gly	Ala	Ser	Val				Variable
110	Ser	Gln	Leu	Glu	Thr			Variable
134	Leu	Lys	Glu					Non-polar aliphatic to polar
145	Lys	Asp	Asn	Gln				Positive to negative/neutral
181	Lys	Asn	Gln					Positive to neutral
183	Val	Ile						No significant change
**p53**								
11 Notothenioid species-specific substitutions in core region. 5’ end highly variant with only 9 conserved residues in 93 aa. 3’ end highly variant with only 29 conserved residues in 81 aa.								
107	Gln	Asn	Glu	Lys	Ala			No significant change
109	Gln	Arg	His	Glu				Variable
113	Thr	Ser						No significant change
126	Gln	Lys	Ile	Ser				Variable
145	Gly	Lys	Ile	Ser				Variable
148	Ile	Pro						Non-polar aliphatic to small
153	Ile	Met	Val					No significant change
181	Ala	Ser	Thr	Leu				Variable
216	Leu	Pro	Arg					Variable
223	Ile	Met	Phe					No significant change
254	Ala	Gln	Pro	Thr				Variable
257	Leu	Val	Glu					No significant change
264	Leu	Phe						Aliphatic to aromatic
268	Ile	Val						No significant change
299	Thr	Ala	Val					Neutral to non-polar aliphatic

References

Aken BL, Achuthan P, Akanni W, Amode MR, Bernsdorff F, Bhai J, Billis K, Carvalho-Silva D, Cummins C, Clapham P, et al. 2017. Ensembl 2017. Nucl Acids Res 45:D635-D642.

Bateman A, Martin MJ, O'Donovan C, Magrane M, Alpi E, Antunes R, Bely B, Bingley M, Bonilla C, Britto R, et al. 2017. UniProt: the universal protein knowledgebase. Nucl Acids Res 45:D158-D169.

Consensus line symbols: * = absolute conservation, . = up to 50% conservation across the alignment at that position. Dark squares are absolute conservation of amino acids. Light squares are conservative changes and white squares are divergent changes. Antarctic species names are in blue, whilst red name denotes a temperate Notothenioid.

Calmodulin

```
DANRE         1 MADQLTEEQIAEFKEAFSLFDKDGDGTITTKELGTVMRSLGQNPTEAELQDMINEVDADG
ELEEL         1 MADQLTEEQIAEFKEAFSLFDKDGDGTITTKELGTVMRSLGQNPTEAELQDMINEVDADG
CTEID         1 MADQLTEEQIAEFKEAFSLFDKDGDGTITTKELGTVMRSLGQNPTEAELQDMINEVDADG
ONCSP         1 MADQLTEEQIAEFKEAFSLFDKDGDGTITTKELGTVMRSLGQNPTEAELQDMINEVDADG
TORCA         1 MADQLTEEQIAEFKEAFSLFDKDGDGTITTKELGTVMRSLGQNPTEAELQDMINEVDADG
NEOIO         1 MADQLTEEQIAEFKEAFSLFDKDGDGTITTKELGTVMRSLGQNPTEAELQDMINEVDADG
POEFO         1 MADQLTEEQIAEFKEAFSLFDKDGDGTITTKELGTVMRSLGQNPTEAELQDMINEVDADG
ASTMX         1 MADQLTEEQIAEFKEAFSLFDKDGDGTITTKELGTVMRSLGQNPTEAELQDMINEVDADG
TAKRU         1 MADQLTEEQIAEFKEAFSLFDKDGDGTITTKELGTVMRSLGQNPTEAELQDMINEVDADG
ORYLA         1 MADQLTEEQIAEFKEAFSLFDKDGDGTITTKELGTVMRSLGQNPTEAELQDMINEVDADG
XIPMA         1 MADQLTEEQIAEFKEAFSLFDKDGDGTITTKELGTVMRSLGQNPTEAELQDMINEVDADG
PARCH         1 MADQLTEEQIAEFKEAFSLFDKDGDGTITTKELGTVMRSLGQNPTEAELQDMINEVDADG
consensus     1 ************************************************************

DANRE        61 NGTIDFPEFLTMMARKMKDTDSEEEIREAFRVFDKDGNGYISAAELRHVMTNLGEKLTDE
ELEEL        61 NGTIDFPEFLTMMAKKMKDTDSEEEIREAFRVFDKDGNGYISAAELRHVMTNLGEKLTDE
CTEID        61 NGTIDFPEFLTMMARKMKDTDSEEEIREAFRVFDKDGNGYISAAELRHVMTNLGEKLTDE
ONCSP        61 NGTIDFPEFLTMMARKMKDTDSEEEIREAFRVFDKDGNGYISAAELRHVMTNLGEKLTDE
TORCA        61 NGTIDFPEFLTMMARKMKDTDSEEEIREAFRVFDKDGNGYISAAELRHVMTNLGEKLTDE
NEOIO        61 NGTIDFPEFLTMMARKMKDTDSEEEIREAFRVFDKDGNGYISAAELRHVMTNLGEKLTDE
POEFO        61 NGTIDFPEFLTMMARKMKDTDSEEEIREAFRVFDKDGNGYISAAELRHVMTNLGEKLTDE
ASTMX        61 NGTIDFPEFLTMMARKMKDTDSEEEIREAFRVFDKDGNGYISAAELRHVMTNLGEKLTDE
TAKRU        61 NGTIDFPEFLTMMARKMKDTDSEEEIREAFRVFDKDGNGYISAAELRHVMTNLGEKLTDE
ORYLA        61 NGTIDFPEFLTMMARKMKDTDSEEEIREAFRVFDKDGNGYISAAELRHVMTNLGEKLTDE
XIPMA        61 NGTIDFPEFLTMMARKMKDTDSEEEIREAFRVFDKDGNGYISAAELRHVMTNLGEKLTDE
PARCH        61 NGTIDFPEFLTMMARKMKDTDSEEEIREAFRVFDKDGNGFISAAELRHVMTNLGEKLTDE
consensus    61 **************.************************.********************

DANRE       121 EVDEMIREADIDGDGQVNYEEFVQMMTAK
ELEEL       121 EVDEMIREADIDGDGQVNYEEFVQMMTAK
CTEID       121 EVDEMIREADIDGDGQVNYEEFVQMMTAK
ONCSP       121 EVDEMIREADIDGDGQVNYEEFVQMMTAK
TORCA       121 EVDEMIREADIDGDGQVNYEEFVQMMTAK
NEOIO       121 EVDEMIREADIDGDGQVNYEEFVQMMTAK
POEFO       121 EVDEMIREADIDGDGQVNYEEFVQMMTAK
ASTMX       121 EVDEMIREADIDGDGQVNYEEFVQMMTAK
TAKRU       121 EVDEMIREADIDGDGQVNYEEFVQMMTAK
ORYLA       121 EVDEMIREADIDGDGQVNYEEFVQMMTAK
XIPMA       121 EVDEMIREADIDGDGQVNYEEFVQMMTAK
PARCH       121 EVDEMIREADIDGDGQVNYEEFVTMMMSK
consensus   121 ***********************.**..*
```

Superoxide dismutase 1 (SOD1)

```
XIPHE        1 MVLKAVCVLKGAGETTGTVHFEQEIESAPVKVTGEISGLTPGDHGFHVHAFGDNTNGCIS
XIPMA        1 MVLKAVCVLKGAGETTGTVHFEQENESAPVKVTGEISGLTPGDHGFHVHAFGDNTNGCIS
POEFO        1 MVLKAVCVLKGAGETTGTVHFEQENESAPVKVTGEIGGLTPGEHGFHVHAFGDNTNGCIS
TAKRU        1 MAMKAVCVLKGAGDTSGTVYFEQENESAPVKLTGEIKGLTPGEHGFHVHAFGDNTNGCIS
DANRE        1 MVNKAVCVLKGTGEVTGTVYFNQEGEKKPVKVTGEITGLTPGKHGFHVHAFGDNTNGCIS
HYPMO        1 MVNKAVCVLKGDGQVTGTVYFEQEAEKSPVKLSGEITGLTAGKHGFHVHAFGDNTNGCIS
SALSA        1 MALKAVCVLKGTGEVTGTVFFEQEGDGAPVKLTGEIAGLTPGEHGFHVHAFGDNTNGCMS
ONCMY        1 MAMKAVCVLKGTGEVTGTVFFEQEGADGPVKLIGEISGLAPGEHGFHVHAYGDNTNGCMS
GADMO        1 MVLKAVCVLKGTGDVTGTVFFEQEGDGAPVKLSGQIAGLAAGEHGFHVHVFGDNTNGCIS
ASTMX        1 MVHKAVCVLKGTGEVTGTVFFEQVGDGAPVKVSGEITGLTPGLHGFHVHAFGDNTNGCIS
CHIHA        1 ---KAVCVFKGAGEASGTVFFEQETDSCPVKLTGEIKGLTPGEHGFHVHAFGDNTNGCIS
TREBE        1 ---KAVCVFKGTGEASGTVFFEQENDSAPVKLTGEIKGLTPGEHGFHVHAFGDNTNGCIS
NEOIO        1 MVIKAVCVLKGAGEASGTVFFEQENDSSPVKLTGEIKGLTPGEHGFHVHAFGDNTNGCIS
GASAC        1 MVVKAVCVLKGAGETTGTIYFEQESDKAAVKLTGEIKGLTPGEHGFHVHAFGDNTNGCIS
LEPOC        1 MVLKAVCVLKGSGEVSGTVHFEQQNGDAPVKVTGKISGLTPGDHGFHVHAFGDNTNGCVS
ORYLA        1 MVLKAVCVLKGTGETNGVVNFEQESDSAPVKVTGEIKGLTPGKHGFHIHVYGDNTNGCVS
ORENI        1 MVLKAVCVLKGTGDTSGTVYFEQENESAPVKLTGEIKGLTPGEHGFHVHAFGDNTNGCIS
consensus    1 ...*****.**.*. .*...*.*. ....**..*.* **..*.****.*..*******.*

XIPHE       61 AGPHYNPFTKNHGGPTDVERHVGDLGNVTAGADNIAKIDIKDTFIKLSGPNSIIGRTMVI
XIPMA       61 AGPHYNPFTKNHGGPTDVERHVGDLGNVTAGADNIAKIDIKDTFIKLSGPNSIIGRTMVI
POEFO       61 AGPHYNPFSKKHGGPTDEERHVGDLGNVTAGADNIAKIDIKDSFIKLSGPNSIIGRTMVI
TAKRU       61 AGPHYNPHNKTHAGPTDADRHLGDLGNVTAGADNIAKIDIKDSMLTLTGPYSIIGRTMVI
DANRE       61 AGPHFNPHDKTHGGPTDSVRHVGDLGNVTADASGVAKIEIEDAMLTLSGQHSIIGRTMVI
HYPMO       61 AGPHFNPYSKNHGGPTDSERHVGDLGNVTAGENGVAKIDIVDKMLTLSGPDSIIGRTMVI
SALSA       61 AGPHFNPHNHTHGGPTDTVRHVGDLGNVTAAADSVAKINIQDEILSLAGPHSIIGRTMVI
ONCMY       61 AGPHFNPHNQTHGGPTDAVRHVGDLGNVTAGADNVAKINIQDKMLTLTGPDSIIGRTMVI
GADMO       61 AGPHFNPHKKDHAGPNDVDRHVGDLGNVTAGADKVAKIDITDKMLSLNGPFSIVGRTMVI
ASTMX       61 AGPHYNPHNKTHGGPTDEIRHVGDLGNVTAGADGVAKINIEDKMLSLSGPHSIVGRTMVI
CHIHA       58 AGPHFNPHNKTHAGPTDENRHVGDLGNVTAAADNVAKLDITDKMITLAGQYSIIGRTMVI
TREBE       58 AGPHFNPHNKTHAGPTDEDRHVGDLGNVTAAADNVAKLNITDKMITLAGQYSIIGRTMVI
NEOIO       61 AGPHFNPHNKTHAGPTDENRHVGDLGNVTAAADNVAKLDITDKMITLAGQYSIIGRTMVI
GASAC       61 AGPHFNPHSKTHAGPNDEIRHVGDLGNVTAGGDNIAKIDITDKVITLTGQHSIIGRTMVI
LEPOC       61 AGPHYNPHNKTHGGPRDENRHVGDLGNVTAGEDKVANINIEDKHITLTGQFSIIGRTMVI
ORYLA       61 AGPHFNPYNKNHGGPEDAERHVGDLGNVTAGDNNVAKIDITDKLIRLSGPDSIVGRTVVV
ORENI       61 AGPHFNPYNKNHGGPKDAERHVGDLGNVTAADN-VAKIEITDKVITLTGPDSIIGRTMVI
consensus   61 ****.**....*.**.* .**.********.....*...* *....*.*. **.***.*.

XIPHE      121 HEKADDLGKGGNEESLKTGNAGGRLACGVIGITQ------
XIPMA      121 HEKADDLGKGGNEESLKTGNAGGRLACGVIGIAQ------
POEFO      121 HEKADDLGKGGNEESLKTGNAGGRLACGVIGITQ------
TAKRU      121 HEKADDLGKGGNEESLKTGNAGGRLACGVIGITQ------
DANRE      121 HEKEDDLGKGGNEESLKTGNAGGRLACGVIGITQ------
HYPMO      121 HEKEDDLGKGNNEESLKTGNAGGRLACGVIGIAQ------
SALSA      121 HEKADDLGKGDNEESRKTGNAGSRLACGVIGIAQ------
ONCMY      121 HEKADDLGKGGNEESLKTGNAGGRQACGVIGIAQ------
GADMO      121 HEKADDLGKGGNEESLKTGNAGSRLACGVIGI--------
ASTMX      121 HEKEDDLGKGGDEESLKTGNAGARLACGVIGIAQ------
CHIHA      118 HEKADDLGKGGNDESLKTGNAGGRLACGVIGIAQMDRRPT
TREBE      118 HEKADDLGKGGNDESLKTGNAGGRLACGVIGIAQ------
NEOIO      121 HEKADDLGKGGNDESLKTGNAGGRLACGVIGIAQ------
GASAC      121 HEKADDLGKGGNEESLKTGNAGSRLACGVIGIAQ------
LEPOC      121 HEKADDLGKGGDDESLKTGNAGGRLACGVIGIAQ------
ORYLA      121 HEKVDDLGKGGNDESLKTGNAGARLACGVIGIAQ------
ORENI      120 HEKVDDLGKGGNEESLKTGNAGGRLACGVIGITQ------
consensus  121 ***.******...**.******.*.*******..
```

Neuroglobin

```
DANRE        1 MEKLSEKDKGLIRDSWESLGKNKVPHGIVLFTRLFELDPALLTLFSYSTNCGDAPECLSS
ASTMX        1 MEKLTGKDKELIRDSWESLGKNKVPHGIVMFTRLFELDPSLLTLFNYKTNCGVVPECLSS
POEFO        1 MGELSVKDKELIRGSWESLGKNKVPHGVIMFSRLFELDPALLNLFHYSTNCDSKQDCLSS
XIPMA        1 MGELSVKDKELIRGSWESLGKNKVPHGVIMFSRLFELDPALLNLFHYSTNCDSKQDCLSS
ORYLA        1 MEKLSGKDKELIRGSWESLGKNKVPHGVIMFSRLFELDPALLSLFNYNTNCGSTQDCLSS
TAKRU        1 MEKLSSKDKELIRGSWDSLGKNKVPHGVIMFSRLFELDPELLSLFHYTTNCGSTQDCLSS
TETNG        1 MEKLSSKDKELIRGSWDSLGKNKVPHGVILFSRLFELDPELLNLFHYTTNCGSTQDCLSS
PARCH        1 ------------------------------------------------------------
PSEGE        1 ---------------------------VFVCGRLFELDPELLTLFHYTTNCGSTQDCLSS
CHAAC        1 MEKLSEKDKELIRGSWESLGKNKVPHGVVMFSRLFELDPELLTLFHYTTNCGSTQDCLSS
DISMA        1 MEKLSEKDKELIRGSWESLGKNKVPHGVVMFSRLFELDPELLTLFHYTTNCGSTQDCLSS
CHIMY        1 MEKLSGKDKELIRGSWESLGKNKVPHGVVMFSRLFELDPELLTLFHYTTNCGSTQDCLSS
NEOIO        1 MEKLSGKDKELIRGSWESLGKNKVPHGVVMFSRLFELDPELLTLFHYTTNCGSTQDCLSS
GYMAC        1 MEKLSGKDKELIRGSWESLGKNKVPHGVVMFSRLFELDPELLTLFHYTTNCGSTQDCLSS
BOVVA        1 MEKLSGKDKELIRGSWESLGKNKVPHGVVMFSRLFELDPELLTLFHYTTNCGSTQDCLSS
ONCMY1       1 MEKLTEKEKELIRVSWESLGKDKVPHGVIMFSRLFELEPALLNLFHYNTNCGTIQDCLSS
ONCMY2       1 MEKLTEKDKELIRGSWESLGKNKVPHGVVMFSRLFELEPALLNLFHYNTNCSPTQDCLSS
LEPOC        1 MEKLTAKDKEMIRDSWERLGKNKLTHGTIMFTRLFELDPELLGLFHYNTPYSSPQECLSS
consensus    1 ..... .......................................................

DANRE       61 PEFLEHVTKVMLVIDAAVSHLDDLHTLEDFLLNLGRKHQAVGVNTQSFALVGESLLYMLQ
ASTMX       61 PEFLEHVTKVMLVVDAAVSHLDDLHTLEDFLLNLGKKHQAVGVNTQSFALVGESLLYMLQ
POEFO       61 PEFLDHVTKVMLVIDAAVSHLDDLHSLEDFLLNLGRKHQAVGVSTQSFTEVGESLLYMLQ
XIPMA       61 PEFLDHVTKVMLVIDAAVSHLDDLHSLEDFLLNLGRKHQAVGVSTQSFTVVGESLLYMLQ
ORYLA       61 PEFLDHVTKVMLVIDAAVNHLDDLHSLEDFLLNLGRKHQAVGVSTQSFAVVGESLLYMLQ
TAKRU       61 PEFLEHVTKVMLVIDAAVSHLDDLHSLEDFLLNLGRKHQAVGVNPQSFATVGESLLYMLQ
TETNG       61 PEFLEHVTKVMLVIDAAVSHLDDLHSLEDFLLNLGRKHQAVGVKPQSFAMVGESLLYMLQ
PARCH        1 ---------VMLVIDAAVSHLDDLPSLEDFLLNLGRKHQAVGVNTQSFAEVGESLLYMLQ
PSEGE       34 PEFLEHVTKVMLVIDAAVSHLDDLPSLEDFLLNLGRKHQAVGVNTQSFAEVGESLLYMLQ
CHAAC       61 PEFLEHVTKVMLVIDAAVSHLDDLPSLEDFLLNLGRKHQAVGVNTQSFAEVGESLLYMLQ
DISMA       61 PEFLEHVTKVMLVIDAAVSNLDDLPSLEDFLLNLGRKHQAVGVNTQSFAEVGESLLHMLQ
CHIMY       61 PEFLEHVTKVMLVIDAAVSHLDDLPSLEDFLLNLGRKHQAVGVNTQSFAEVGESLLYMLQ
NEOIO       61 PEFLEHVTKVMLVIDAAVSHLDDLPSLEDFLLNLGRKHQAVGVNTQSFAEVGESLLYMLQ
GYMAC       61 PEFLEHVTKVMLVIDAAVSHLDDLPSLEDFLLNLGRKHQAVGVNTQSFAEVGESLLYMLQ
BOVVA       61 PEFLEHVTKVMLVIDAAVSNLDDLPSLEDFLLNLGGKHQAVGVNTQSFAEVGESLLYMLQ
ONCMY1      61 PEFLDHVTKVMLVIDAAVSHLDNLHTLEDFLLNLGKKHQAVGVNTQSFAVVGESLLYMLQ
ONCMY2      61 PEFLDHVTKVMLVIDAAVSHLDDLHTLEDFLLNLGKKHQAVGVNTQSFAVVGESLLYMLQ
LEPOC       61 PEFVDHINKVMLVVDAAVTHLDNLHSLEEYLVNLGRKHQAVGVQTQSFAAVGESLLYMLE
consensus   61 .........****.****..**.*..**..*.***.*******..***..******.**.

DANRE      121 SSLGPAYTTSLRQAWLTMYSIVVSAMTRGWAKNGEHKSN
ASTMX      121 CSLGTAYTTALRQAWLNMYTIVVSAMTRGWAKNGEHKSN
POEFO      121 CSLGQAYTAPLRQAWLNMYSIVVAVMSRGWAKNGEDKAD
XIPMA      121 CSLGQAYTAPLRQAWLNMYTIVVAVMSRGWSKNGEDKAD
ORYLA      121 CSLGQAYTAALSQAWLNMYSIVVAAMSRGWAKNGEDKAD
TAKRU      121 CSLGQAYTASLRQAWLNMYSVVVAAMSRGWAKNGEDKAD
TETNG      121 CSLGQAYTASLRQAWLNMYSVVVASMSRGWAKNGEDKAD
PARCH       52 CSLGQAYTAPLRQAWLNLYSIVVAAMSQGWAKNGEDKAD
PSEGE       94 CSLGQAYTAPLRQAWLNLYSIVVAAMSQGWAKNGEDKAD
CHAAC      121 CSLGQAYTAPLRQAWLNLYSIVVAAMSQGWAKNGEDKAD
DISMA      121 CSLGQAYTAPLRQAWLNLYSIVVAAMSQGWAKNGEDKAD
CHIMY      121 CSLGQAYTAPLRQAWLNLYSIVVAAMMPRLG--------
NEOIO      121 CSLGQAYTAPLRQAWLNLYSIVVAAMMPRLG--------
GYMAC      121 CSLGQAYTAPLRQAWLNLYSIVVAAMMPRLG--------
BOVVA      121 CSLGQAYTAPLRQAWLNLYSIVVAAMSRGWA--------
ONCMY1     121 CSLGQGYTAPLRQAWLNMYTIVVAAMSRGWAKNGEHKTD
ONCMY2     121 CSLGHGYTGPLRQAWLNMYTIVVAAMSRGWAKNGEHKTD
LEPOC      121 RSLGPAYTSTLRHAWLTLYSVVVEAMSSGWTKNGKSQTD
consensus  121 .***..**..*..***..*..**..*.............
```

Dihydrofolate reductase

```
TAKRU        1 MSRVVNAIVAVCPDLGIGMNGDLPWHPIRLNKEFVHFRKMTSTPSVNGKQNVVIMGRKTW
TETNI        1 MARVLNAIVAVCPDLGIGRNGDLPWHPIRLDNEFKHFRKMTSTPSVNGKQNVVIMGRKTW
HARAN        1 MSRMLNAIVAVCPDLGIGMDGQLPWHPVRLNNEFKHFRKITATPSVEGKQNVVIMGRKTW
NEOIO        1 MSRMLNAIVAVCPDLGIGMDGQLPWHPVRLNNEFKHFRKMTATPSVEGKQNVVIMGRKTW
GASAC        1 MSRVLNGIVAVCPDLGIGCHGNLPWHPLRLSNEFKHFRRMTATASVKDKQNVVIMGRKTW
ORENI        1 MPRVLNAIVAVCPDRGIGNKGNLPWHPIRLSKEFAHFRKMTATPSVKGKQNVVIMGKKTW
LEPOC        1 MPRPINCIVAVCPNMGIGHNGNLPWHPKRLSNEFKYFQKMTMTPTLEGQQNAVIMGRKTW
DANRE        1 MSRILNCIVAVCPDMGIGKNGNLPWHPIRLSNELKHFQKMTMTPSDEGKKNVVIMGRKTW
consensus    1 *.*..*.******..*** .*.*****.**..*...*...*.*.......*.****.***

TAKRU       61 FSIPERNRPLANRINIVLSRRCREPPAGAHHLARDLPSALRLVDS-ELAEQADQVWVIGG
TETNI       61 FSIPEKHRPLANRINIVLSRRSREPPAGAHHLAHDLSSALQLVET-QLADR-DQVWVIGG
HARAN       61 FSIPEKNRPLNNRINIVLSRECRAPPAGAHHLAPDFNSALRLVET-ELAERTDQVWVIGG
NEOIO       61 FSIPEKNRPLNNRINIVLSRECRAPPAGAHHLAPDFSSALRLVET-ELAERTAQVWVIGG
GASAC       61 FSIPEKNRPLNNRINIVLSRELKAPPAGAHHLAPDFSSALRLVDT-ELAEQADQVWVIGG
ORENI       61 YSIPEKNRPLSNRINIVLSRECKVPPAGAHYLASDFSSALRLIDT-ELADQADQVWVIGG
LEPOC       61 FSIPERNRPLKNRINIVLSRELKNPPEGAHYLASDFSSALRLLDSAPQTDQVDQVWVIGG
DANRE       61 FSIPAAHRPLKNRINIVLSRELKTAPEGAHYLASDFSSALHLLDSGELEKLVDQVWIIGG
consensus   61 .***...*** *********... .*.***.** *..***.*... ..... .***.***

TAKRU      120 SSLYQELMEGTGTTRLFVTRILKQFECDTFLPEINPARYRRLPQFPGVAQELQEEEGIQY
TETNI      119 SSLYQEMMERPGTSRLFVTHVLKQFDCDTFLPEIRPQRYRLLPQFPGVPQELQEEKGIQY
HARAN      120 SSLYKELMQFPGTRRLFITRIMKQFECDTFLPEISLDKYRLLPQFPGVPHELQEENGIQY
NEOIO      120 SSLYKELMQFPGTRRLFVTRIMKQFECDTFLPEISLDRYRLLPQFPGVPHELQEENGIQY
GASAC      120 SSLYKELLESPGTRRLFVTRILKQFECDTFLPEICPDRYRLLPEFPGVPEELQEENGVQY
ORENI      120 SSLYKEMMGSTGMRRLFVTQILKQFECDTFLPEISLDKYRLLPEFPDVPQELQEENGIQY
LEPOC      121 SSVYKEAMEAPGWCRLFVTQILQDFECDTFLPEINLNKYRLLPEFPGVPLEVQEENGIRY
DANRE      121 SSLYKEVMERSGHRRLFVTRILKQFDCDTFIPNFDMDKYKLLPEFPGVPVGLQEDNGVQY
consensus  121 **.*.*... .*..***.*.....*.****.*.. ...*..** **.*. ..**..*..*

TAKRU      180 RFQVYESIEH------
TETNI      179 RYQVYESLE-------
HARAN      180 RFEVYESIDELC----
NEOIO      180 RFEVYESIDELCNLNK
GASAC      180 RFEVYESIDNK-----
ORENI      180 RFKVYESIQE------
LEPOC      181 KFEVYESIQN------
DANRE      181 VFEVYESIEH------
consensus  181 ...****..
```

Dihydrofolate reductase-like

```
HARAN         1 MENTQNKVQRKPVRLIAAACNDMGIGKEGGMPWSLPSEFQWFLNKVTTVSRPGKFNMMVW
PARCH         1 MENTQNKVQRKPVRIIAAACNDMGIGKEGGMPWSLPSEFQWFLNKVTTVSRPGKFNMMVW
NEOIO         1 -------------------------------------SEFQSFLNRVTTVSRPGKFNMMVW
GASAC         1 --MSGDNVQKKPVRLIAAACSGGGIGKDGQMPWDLPSEFRYFRNHVQGVSRPGKMNMMVW
TAKRU         1 --MEAAKGNKKPVRVIAAVCNNRGIGKDNQMPWSIPAEFQYFLNTVTRVSRPGNMNMMVW
TETNG         1 --MEPAKVIKKPVRLIAAKCNGGGIGKDNRMPWSLPSEFRFFLSTITRVSRPGKMNMIIW
POEFO         1 MEKDREKVQKKPVRLIAATCNNMGMGKDGTLPWSLPSEFQYFLNTITRVSRPGNMNLLIW
XIPMA         1 MEQDRETVRKKPVQLIAAICNNMGMGKDGTLPWSLPSEFQYFLNTITRVSRPGNMNLLIW
ORYLA         1 MEN-RLEERRKPVRLIAAVCRNNGIGKDGTLPWSLPSEFQYFLNTITRVSRPGNVNLMIW
DANRE         1 MMNIEGEEMRKPIRLIAAACRDMGIGKDGQIPWCLPKEFQFLLDTITAVSAPGKKNLIVW
LEPOC         1 MSDCEGKVHRKSIRLIAAACNGMGIGKDGILPWSLPKEFKFFLDTITSVSSPDKKNLLIW
consensus     1 .     .. ......... .. ....... .......**........ **.*...*...*

HARAN        61 GKKSWFNHPESTFPLPNTLHAVLSLTLDSPPDHAHFVGSDLEAAVRLAGSPPLADLIETI
PARCH        61 GKKCWFSHPESTFPLPNTLHAVLSLTLDSPPDHAHFVCSDLEAAVRLAGSPPLADLIETI
NEOIO        25 GKTCWFSHPESTFPLPNTFHAVLSLTLDSPPDHAHFVCSDLEAAVCLAGSPPLADLIETI
GASAC        59 GKLCWYSVPKPDFPLPNVLHVVLSKTLESVPDHAHFLCEDLDAAARLAVQPPLADLIETI
TAKRU        59 GKQCWVSHPDSTFPLPNILHAVLSKTLFTVPDHAHFLCESLDAAVRLASEPPLADLIEII
TETNG        59 GKQCWISHPESTFPLPNVLHTVLSTTLFAVPDHAHFVCETLDAAVRLASEPPLADLIEIV
POEFO        61 GRLCWNSHPENIFPLANSLHVVLSKTLSSVPDHAHFLCQDFESAVRLAAQPPLSGIIETV
XIPMA        61 GRLCWNSHSENMFPLANSLHVVLSKTLSSAPDHAHFLCQDFESAVRLAAQPPLSDIIETV
ORYLA        60 GRLCWFSHPDDLFPLPNSLHVVLSKTLTSVPNHAQFLCGDFESAVRLAALPPLADIIETI
DANRE        61 GRICWFSCPETVFPLANCINLVLSRKMISVPPHAHYLCKDFDSIIRLVSEPPLCHTVEVI
LEPOC        61 GKRCWISFPESLHPLANCIHVVLSRTMGCVPDHAHYLCHDLPSVIQLGSTHPLSDKIETI
consensus    61 *. .*...... .**.*....***... ..*.**.... .......*.. .**....*..

HARAN       121 WIVGGMQVYKEALLHQWCDLVYLTKVMADFDCDVFFPEFDRELFKLQEGFPGVPSEIQEE
PARCH       121 WIVGGVQVYKEALLHRWCDLVYLTKVMADFDCDVFFPKFDRELFKLQEG-----------
NEOIO        85 WIVGGVQVYKEALLHRWCDLVYLTKVMADFDCDVFFPEFDRELFKLQEGFPGVPTEIQEE
GASAC       119 WVVGGTQVYEVALKHPWCDLVYLTDVMADFDCNVFFPEFDRGLFKVQEGFPGVPSEIQEE
TAKRU       119 WIVGGVQVYKEAMEHPRCDLIYLTDIMAEFECDVFFPEFDKKLFEVQDSFPDVPNGIQED
TETNG       119 WIVGGVQVYKEAMEHPWCDLIYLTDIMAEFECDVFFPEFDRQLFQVQDGFPDVPDGIQEE
POEFO       121 WILGGTQVYEDALKHPWCDLLYLTDVMADFDCDVFFPDFDRELFKLQETFPDVPIEIQEE
XIPMA       121 WILGGTQVYEDALKHPWCDLLYLTDVMADFDCDVFFPEFDRELFKLQEKFPDVPSEIQEE
ORYLA       120 WVLGGTKVYEEALKHPCCDLLYFTDVMADFDCDVFFPDFDRELFRVQEEFPDVPSEVQEE
DANRE       121 WILGGTEVYKESLEHPWCDLIYLTNIMANFECDVFFPEFDPNIFRKQKSFPGVPDEIIEE
LEPOC       121 WILGGAELYKESLKHPWCDYIYLTQVMADFDCDTFFPEFDREIYKLQDEFPGVPSEIQED
consensus   121 *..** ..*.... *..**..*.*..**.*.*..***.**......*. .. ........

HARAN       181 KGVKYQFQVFKRETGDNV-
PARCH           ------------------
NEOIO       145 KGVKYQFQVFKRETGNKV-
GASAC       179 NGIKYKFQVFKKETGDAV-
TAKRU       179 NGIKYKCQVYKRKTAVCLL
TETNG       179 NGIKYKCQVYKRETAAEDQ
POEFO       181 NGIRFKCQVFKKKTDDTM-
XIPMA       181 NGIRFKCQVFKKKTDDAF-
ORYLA       180 NGIKFKCQVFKRVTMEDHL
DANRE       181 NGIKFQFQVFKKIKN----
LEPOC       181 QGIKLKFQVFKKDMH----
consensus   181 ...... ..... .
```

P53

```
HARAN        1 MEEQSLDDLTLSQTMPLSQDSFSELWNTVSAPLFSYLTPTVINVPEETWKTDGHMDMLLL
NEOIO        1 MEEQSLDDLTLSQTMPLSQDSFKELWNTVSAPLFSYLTPTVN-VPEETWKTDGNMDMLLL
TAKRU        1 MEDE-------GFSLPLSQDTFQDLWENVDVCMHP--DSPVS------------QLMNYP
TETNG        1 MEEE-------TFSLPLSQDTFQDLWENVAAPSISTIQTTVSGNECW---QDGSLTMALM
POEFO        1 -MET-------NFPLPLSQDTFHELWNTVVLSTENESLATGD---------------GLL
XIPMA        1 MEEA-------DLTLPLSQDTFHDLWNNVFLSTENESLPPPE---------------GLL
consensus    1 ...        ....*****.*..**. * ..  . . ...          .. . ....

HARAN       61 NDHSLNEVFDEKLFELPPPDMSTMDGVNPTSSTVPVTSDYPGEYGFQLQFQKTGTAKSVT
NEOIO       60 NDHSLTEVFDEELFELPPPDMSTMDGVNPTSSTVPVTSDYPGEYGFQLQFQKTGTAKSVT
TAKRU       40 ELP-----FNEELFNLP-SEMASKDSANLSTPTVPVTTDYPGEYGFELRFQKSGTAKSVT
TETNG       51 DMP-----YDEDLFNLP-SELPNKDGANSSCPTVPVTTDHPGEYDFKLRFQKSGTAKSVT
POEFO       38 DLN-------MDFWENGELPQQETKNV-PAAPMVPAISNYAGELDFALHFNDSGTAKSVT
XIPMA       39 SQN-------MDFWEDPET-MQETKNV-PTAPTVPAISNYAGEHGFNLEFNDSGTAKSVT
consensus   61 ..      ......... ... . .........**......**..*.*.*...*******

HARAN      121 STYSEQLNKLYCQLAKTTPVEVLFGKEIPLGAIIRATAVYKKTEHVAEVVRRCPHHQNED
NEOIO      120 STYSEQLNKLYCQLAKTTPVEVLLGKEIPLGAIIRATAVYKKTEHVAEVVRRCPHHQNED
TAKRU       94 STYSEILNKLYCQLAKTSLVEVLLIKKPPAGAVLRATAIYKKIEHVADVVRRCPHHQNED
TETNG      105 STYSESLNKLYCQLAKTSPLEVLLSREPPLGAMLRATAIYKKTEHVAEVVRRCPHHQNED
POEFO       90 STFSEKLTKLFCQLAKTTPIGILVKVEPPQGAVIRATAVYKKTEHVAEVVKRCPHHQSED
XIPMA       90 STYSVKLGKLFCQLAKTTPIGVLVKEEPPQGAVIRATAVYKKTEHVGEVVKRCPHHQSED
consensus  121 **.*. *.**.******.....*. ...*.**..****.***.***..**.******.**

HARAN      181 AADHRSHLIRVEGSQRAQYFEDPNTKRQSVTVPYELPQLGSEITTILLSFMCNSSCMGGM
NEOIO      180 AADHRSHLIRVEGSQRALYFEDTNTKRQSVTVPYELPQLGSEITTILLSFMCNSSCMGGM
TAKRU      154 SAAHRSHLIRMEGSQRAQYFEDPHTKRQSVTVPYEPPQLGSEFTTILLSFMCNSSCMGGM
TETNG      165 STENRSHLIRMEGSQRAQYFEDPHTKRQSVTVPYEPPQLGSEFTTILLSFMCNSSCMGGM
POEFO      150 TSDNKSHLIRVEGSQLAQYFEDPNTKRQSVTVPYERPQRGSEMTTILLSFMCNSSCMGGM
XIPMA      150 LSDNKSHLIRVEGSQLAQYFEDPNTRRHSVTVPYERPQLGSEMTTILLSFMCNSSCMGGM
consensus  181 ... .*****.****.*.****..*.*.******* **.***.*****************

HARAN      241 NRRPILTILTLETAEGLVLGRRCLEVRICACPGRDRKTEEENSTKVQSGTKQTKKRKSTP
NEOIO      240 NRRPILTILTLETAEGLVLGRRCLEVRICACPGRDRKTEEENSTKMQSGTKQTKKRKSTP
TAKRU      214 NRRPILTILTLETQEGVVLGRRCFEVRVCACPGRDRKTEEANSTNMQNGTKETKKRKSVP
TETNG      225 NRRPILAILTLETQEGVVLGRRCFEVRVCACPGRDRKTEEANSTKMQTETKDAKKRKSAP
POEFO      210 NRRPILTILTLETPEGEVLGRRCFEVRVCACPGRDRKTEEEN--LEKNGTKQTKKRKSAP
XIPMA      210 NRRPILTILTLETTEGEVLGRRCFEVRVCACPGRDRKTEEGN--LEKSGTKQTKKRKSAP
consensus  241 ******.****** **.******.***.************.*.......**..*****.*

HARAN      301 ---GSSSMKKSRPTSSAEEEDKDVFVLNVRGHERYEMLKKINDGLELLDKDRKPETRVKH
NEOIO      300 ---GSSSMKKSRPTSSAEEEDKDVFVLNVRGRERYEMLKKINDGLELLDKDRKPETRVKH
TAKRU      274 PPAAAAAAKKSKTASSAEEDDKELFTLQIRGRKRYEMLKKINDGLELLENKPKCKAAAKP
TETNG      285 -TSDSTTVKKSRTASSAEEDDKEVFTLQIRGRKRYEMIKRINDGLDLLENKTKSKTTYKP
POEFO      268 -APDTSTAKKSKSVSSGEDEDKELYTLQIRGRERFLMFKKLNDGLELMEKMG---PKKKQ
XIPMA      268 -APDTSTAKKSKSASSGEDEDKEIYTLSIRGRNRYLWFKSLNDGLELMDKTG---PKIKQ
consensus  301    .....***...**.*..**....*..**..*....*..****.*...  .  ...*

HARAN      358 EFALPSSGKRLLHRGDKSDSD
NEOIO      357 EFALPSSGKRLLHKGDKSDSD
TAKRU      334 ECPVPPRGKRLLHRGEKSDSD
TETNG      344 EGPVLPSGKRLMHRGEKSDSD
POEFO      324 EVPAPSSGKRLLKGG--SDSD
XIPMA      324 EIPAPSSGKRLLKGG--SDSD
consensus  361 * .....****...*..****
```

Accession numbers for fish species used in ClustalW alignments of candidate proteins

Abbreviation	Species name	Common name	Calmodulin	DHFR	DHFR-like	Neuroglobin	p53	SOD1
DANRE	*Danio rerio*	Zebrafish	Q6PI52	A2BGQ1	F1QRU7	Q90YJ2		O73872
POEFO	*Poecilia formosa*	Amazon molly	A0A087XTZ7		A0A087YOQ3	A0A087YDQ5	A0A087XP53	A0A087YGV3
ASTMX	*Astyanax mexicanus*	Blind cave fish	W5L960			W5K4Z1		W5KMP4
TAKFU	*Takifugu rubripes*	Fugu (pufferfish)	H2TXN3	H2SSG1	H286U1	H2VBJ2	H2U135	H2T728
ORYLA	*Oryzias latipes*	Medaka	I6L4R5		H2MP65	H2MNQ2		H2LHN5
XIPMA	*Xiphophorus maculatus*	Platyfish	M3ZHJ6		M4AXY9	M4AL53	Q9W679	M4AT47
LEPOC	*Lepistosteus oculatus*	Spotted gar		W5MD25	W5NDT1	W5MYS7		W5MSO0
TETNG	*Tetraodon nigroviridis*	Green spotted pufferfish		H3CG21	H3CSF7	Q90W04	H3CXQ0	
ORENI	*Orechromis niloticus*	Nile tilapia		I3JW88				I3JAL8
GADMO	*Gadus morhua*	Cod						ENSGMOP 00000016337
GASAC	*Gasterosteus aculeatus*	Stickleback		ENSGACG 00000003734	ENSGACG 00000013483			ENSAGP 00000027216
ELEEL	*Electrophorus electricus*	Electric eel	P02594					
CTEID	*Ctenopharyngodon idella*	Grass carp	Q6IT78					

ONCSP	*Oncorhynchus sp.*	Salmon species	P62156					
TORCA	*Torpedo californica*	Pacific electric ray	P62151					
XIPHE	*Xiphophorus helleri*	Green swordtail						D9JOD6
HYPMO	*Hypophthalmichthys molitrix*	Silver carp						D9MPI5
SALSA	*Salmo salar*	Atlantic salmon						Q3ZLR1
ONCMY	*Oncorhynchus mykiss*	Rainbow trout				P59742, P59743		Q8QHI0
**Temperate Notothenioid**								
BOVVA	*Bovichtus variegatus*	Thornfish				D2EA92		
**Antarctic Notothenioids**								
HARAN	*Harpagifer antarcticus*	Plunderfish		This study	This study		This study	
PARCH	*Parachaenichthys charcoti*	Dragonfish	This study		This study	This study		
NEOIO	*Neopagetopsis ionah*	Jonah's icefish	This study	This study	This study	D2EA95	This study	This study
PSEGE	*Pseudochaenichthys georgianus*	South Georgia icefish				This study		
CHIHA	*Chionodraco hamatus*	Crocodile icefish						Q3ZLR2

TREBE	*Trematomus bernacchi*	emerald rockcod						Q3ZLR3
CHAAC	*Chaenocephalus aceratus*	blackfin icefish				P86880		
DISMA	*Dissostichus mawsoni*	Antarctic toothfish				P86881		
CHIMY	*Chionodraco myseri*	Myers' icefish				D2EA94		
GYMAC	*Gymnodraco acuticeps*	ploughfish				D2EA93		
